# A genomic comparison of two termites with different social complexity

**DOI:** 10.3389/fgene.2015.00009

**Published:** 2015-03-04

**Authors:** Judith Korb, Michael Poulsen, Haofu Hu, Cai Li, Jacobus J. Boomsma, Guojie Zhang, Jürgen Liebig

**Affiliations:** ^1^Department of Evolutionary Biology and Ecology, Institute of Biology I, University of FreiburgFreiburg, Germany; ^2^Section for Ecology and Evolution, Department of Biology, Centre for Social Evolution, University of CopenhagenCopenhagen, Denmark; ^3^China National Genebank, BGI-ShenzhenShenzhen, China; ^4^Centre for GeoGenetics, Natural History Museum of Denmark, University of CopenhagenCopenhagen, Denmark; ^5^School of Life Sciences, Arizona State UniversityTempe, AZ, USA

**Keywords:** chemical communication, genomes, immunity, social organization, social insects, symbiosis, termites, transposable elements

## Abstract

The termites evolved eusociality and complex societies before the ants, but have been studied much less. The recent publication of the first two termite genomes provides a unique comparative opportunity, particularly because the sequenced termites represent opposite ends of the social complexity spectrum. *Zootermopsis nevadensis* has simple colonies with totipotent workers that can develop into all castes (dispersing reproductives, nest-inheriting replacement reproductives, and soldiers). In contrast, the fungus-growing termite *Macrotermes natalensis* belongs to the higher termites and has very large and complex societies with morphologically distinct castes that are life-time sterile. Here we compare key characteristics of genomic architecture, focusing on genes involved in communication, immune defenses, mating biology and symbiosis that were likely important in termite social evolution. We discuss these in relation to what is known about these genes in the ants and outline hypothesis for further testing.

## Introduction

The termites are “social cockroaches,” a monophyletic clade (Infraorder “Isoptera”) nested within the Blattodea (Inward et al., 2007a; Engel et al., 2009; Krishna et al., 2013). They superficially resemble the ants in having wingless worker foragers, but are fundamentally different in a series of ancestral traits that affect the organization of their eusocial colonies (Korb, 2008; Howard and Thorne, 2011). The (eu)social Hymenoptera are haplodiploid holometabolous insects whose males develop from haploid eggs and have transient roles in social life, because they survive only as sperm stored in the spermatheca of queens. Hymenopteran colonies thus consist of female adults that develop from fertilized eggs to differentiate into workers, virgin queens and occasionally soldiers of which only the former care for the helpless grub-like larvae. By contrast, termites are diploid hemimetabolous insects whose colonies usually have workers, soldiers, and reproductives of both sexes. Both have life-time monogamy upon colony founding as ancestral state (Hughes et al., 2008; Boomsma, 2013), but in contrast to the eusocial Hymenoptera, royal pairs regularly remate to produce immatures that increasingly come to resemble the workers, soldiers, and reproductives into which they differentiate. Hence, termite caste differentiation is based on phenotypic plasticity among immatures (Korb and Hartfelder, 2008; Miura and Scharf, 2011), while the eusocial Hymenoptera have castes of adults (Wilson, 1971).

Termites and ants also share many traits that convergently evolved in response to similar selective pressures (Thorne and Traniello, 2003; Korb, 2008; Howard and Thorne, 2011). Both are mostly soil-dwelling and thus continuously exposed to high pathogen loads and their long-lived, populous and genetically homogenous colonies appear to be ideal targets for infections (Schmid-Hempel, 1998). However, both the ants and the termites also evolved impressive disease defense strategies, which have implied that very few pathogens have been able to specialize on infecting perennial ant and termite colonies over evolutionary time (Boomsma et al., 2005). In large part this appears to be due to immune defenses operating both at the individual and the collective (social immunity) level (Cremer et al., 2007; Rosengaus et al., 2011). Another common characteristic of the ants and termites is that both evolved complex communication systems that largely rely on chemical cues, such as cuticular hydrocarbons (CHCs), for nestmate recognition and within-colony communication (e.g., Liebig, 2010; Van Zweden and D'Ettorre, 2010). Strikingly, long-chained CHCs of queens often appear to function as fertility signals for workers of both lineages (Liebig et al., 2009; Weil et al., 2009; Liebig, 2010; van Oystaeyen et al., 2014). Here, we offer the first comparative exploration of the extent to which lineage ancestry has determined these convergent phenotypic similarities based on the first two termite genomes that became recently available (Poulsen et al., 2014; Terrapon et al., 2014).

**Figure 1:**
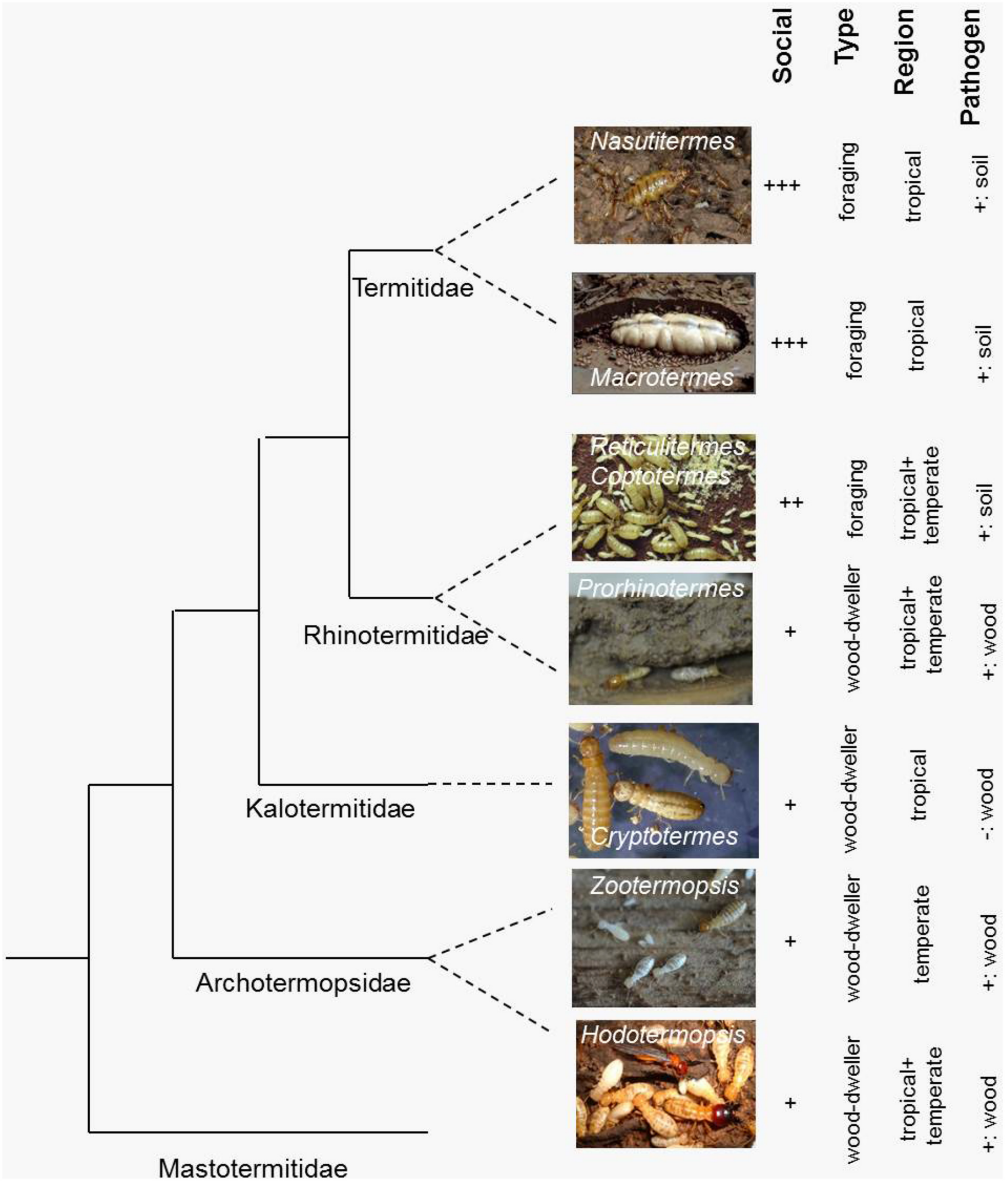
**Simplified phylogeny of the main termite study species with their key traits**. Shown is a cladogram of termite genera on which some genomic/molecular genetic research has been done. Added to the right are characteristic social and ecological traits. Social: increasing social complexity from + to +++ (e.g., increasing colony size, division of labor, morphological differentiation between castes); Type: life type, foraging vs. wood dwelling; Region: temperate vs. tropical; Pathogens: soil pathogens vs. wood-decaying fungi, +, present; −, absent. Study species (photo credits): *Nasutitermes takasagoensis* (Kenji Matsuura), *Macrotermes natalensis* (Judith Korb), *Reticulitermes speratus* (Kenji Matsuura), *Reticulitermes flavipes* (not shown), *Coptotermes formosanus* (not shown), *Prorhinotermes simplex* (Judith Korb), *Cryptotermes secundus* (Judith Korb), *Zootermopsis nevadensis* (Judith Korb), *Hodotermes sjostedti* (Toru Miura).

**Figure 2:**
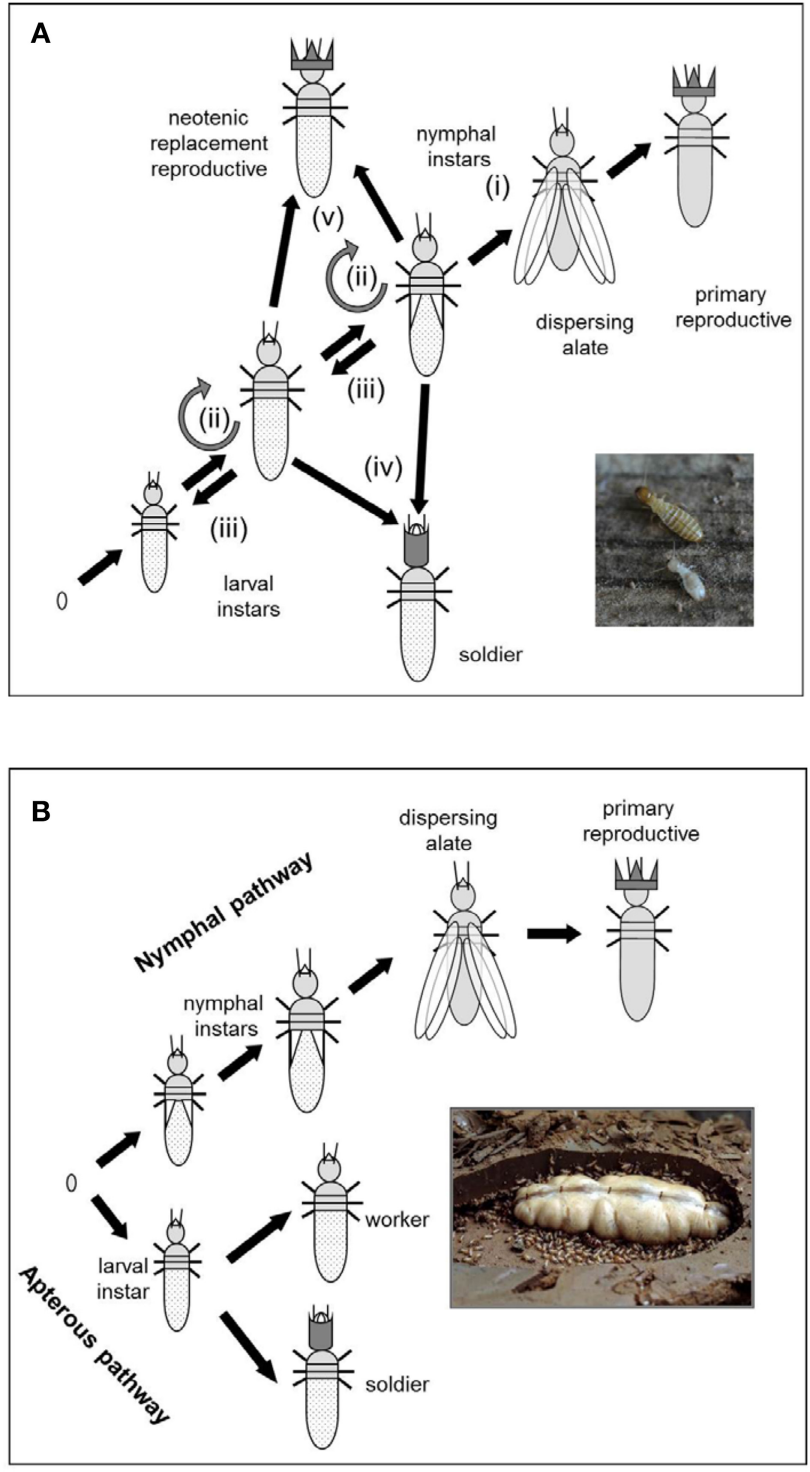
**Developmental pathways of (A) wood-dwelling termites such as *Z. nevadensis* and (B) foraging higher termites such as *M. natalensis***. Wood-dwelling termites have totipotent immature stages that can explore all caste options, whereas higher termites have a bifurcating caste development pathway splitting into a nymphal line leading to winged dispersing alates and an apterous line leading to workers and soldiers. In *M. natalensis* this bifurcation is already established in the egg stage. (i) progressive development via nymphal instar(s) into winged sexuals (alates) that disperse and found a new nest elsewhere; (ii) stationary molt remaining in the same instar; (iii) regressive development into an “earlier” instar (gray semi-circle); (iv) development into a soldier, and (v) development into a neotenic replacement reproductive that reproduces within the natal nest. Part (a) is adapted from Korb et al. (2012b). (Photo credits: Judith Korb).

The two termite genomes represent opposite ends of the social complexity spectrum within the Isoptera (Roisin, 2000) (Table 1) as they exemplify the two fundamental termite life types: the wood-dwelling one-piece nesters and the central place foraging lineages that generally differ in social complexity, feeding ecology, gut symbionts, and developmental plasticity (Abe, 1987; Korb, 2007; Korb and Hartfelder, 2008) (Figure 1). *Zootermopsis nevadensis* belongs to the former type and *Macrotermes natalensis* to the latter. Wood-dwelling species (Abe, 1987; Shellman-Reeve, 1997) nest within a single piece of dead wood that serves both as food and nesting habitat so the termites never leave their nest to forage. This social syndrome is widely considered to be ancestral (e.g., Noirot and Pasteels, 1987, 1988; Inward et al., 2007b) and associated with high degrees of developmental plasticity for the individual termites (Figure 2A). Workers remain totipotent immatures throughout several instars that commonly develop further into sterile soldiers, winged sexuals (alates) that found new nests as primary reproductives, or neotenic reproductives that reproduce within the natal nest (Figure 2A).

**Table 1:** **Summary of traits that differ between the two study species**.

**Traits**	***Z. nevadensis***	***M. natalensis***
Social complexity	Less complex	Highly complex
Life type	Wood-dwelling single-piece nester	Foraging multiple-piece nester
Developmental plasticity	Totipotent workers and a single linear developmental pathway	Restricted developmental options for both workers and reproductives; bifurcated development
Food and digestion	Decaying wood, digested with the help of protists and bacterial gut symbionts	Dead plant material (incl. wood), which is primarily decomposed by symbiotic *Termitomyces* fungi, with additional roles of gut bacteria
Potential pathogen load	Predicted to be high, mainly because the logs inhabited by dampwood termites also harbor many wood-decaying fungi	Predicted to be high, with sources being mainly soil microbes and wood-decaying fungi carried to the nest with the substrate particles
Geographic distribution	Temperate	Tropical and sub-tropical

*Traits 1–3 co-vary in termites in that wood-dwelling termites with totipotent workers are always less socially complex, while foraging termites are more socially complex with workers having restricted developmental options. However, huge trait variability exists within foraging species, see also Figure 1*.

The foraging termite species (also called “multiple piece nesters”; Abe, 1987; Shellman-Reeve, 1997) forage for food outside the nest at some point after colony foundation and bring it back to the colony to feed nestmates. They represent more than 85% of the extant termite species (Kambhampati and Eggleton, 2000). They have true workers and an early separation into distinct developmental pathways (Roisin, 2000; Korb and Hartfelder, 2008) (Figure 2B). In the apterous line, individuals are unable to develop wings and can thus never disperse as reproductives. They become workers and soldiers, but can in some species also advance to become neotenic reproductives in their own nest. In the nymphal line, however, individuals develop wings and dispersing phenotypes that found new colonies elsewhere (Figure 2B). The Macrotermitinae to which *Macrotermes natalensis* belongs are special examples of foraging termites because their colonies are dependent on nutrition provided by a *Termitomyces* symbiont (Basidiomycota: Agaricales) (Wood and Thomas, 1989; Nobre et al., 2011). This fungal symbiosis is evolutionarily derived and comes in addition to more fundamental protist (lower termites) and bacterial gut symbionts (all termites), which have played major roles throughout termite evolution. *Macrotermes* species have two (major/minor) worker castes and two (major/minor) soldier castes (Ruelle, 1970) that may be determined as early as the egg stage (suggested for *Macrotermes michaelseni* by Okot-Kotber, 1985). *Macrotermes* colonies often build conspicuous mounds that may harbor several millions of individuals (Noirot and Darlington, 2000; Korb, 2011).

We compare the genomes of these divergent species (Table 1) with those of other insects and outline first hypotheses how sociality and ecological factors left their footprints in the genomes.

## Materials and methods

### Construction of gene families

To gain insight into the evolution of gene families in termites, we clustered genes from 12 insect genomes (pea aphid: *Acyrthosiphon pisum*: The International Pea Aphid Genomics Consortium, 2010; body louse: *Pediculus humanus*: Kirkness et al., 2010; flour beetle: *Tribolium castaneum*: Richards et al., 2008; fruitfly: *Drosophila melanogaster*: Adams et al., 2000; jewel wasp: *Nasonia vitripennis*: Werren et al., 2010; honeybee: *Apis mellifera*: The Honeybee Genome Sequencing Consortium, 2006; ants: *Acromyrmex echinatior*: Nygaard et al., 2011, *Atta cephalotes*: Suen et al., 2011, *Camponotus floridanus, Harpegnathos saltator*: Bonasio et al., 2010; termites: *Z. nevadensis, M. natalensis*), the water flea *Daphnia pulex* (Colbourne et al., 2011), and the round worm *Caenorhabditis elegans* (Coulson and C. elegans Genome Consortium, 1996). The gene sets of the species that we chose were downloaded from the Ensembl database (Flicek et al., 2014), except for ants and termites which were downloaded from their own reference databases. Then we used Treefam (Li et al., 2006) to construct gene families. For more information see also Terrapon et al. (2014) and Poulsen et al. (2014) (Table S1).

### Functional annotation of termite genes

InterproScan v4.8 (Zdobnov and Apweiler, 2001) was used to annotate motifs and domains of translated proteins in two termites. Protein sequences were searched against SUPERFAMILY, Pfam, PRINTS, PROSITE, ProDom, Gene3D, PANTHER, and SMART databases in Interpro with default parameter settings. GO (gene ontology) terms for each gene were obtained from the Interpro database according to the relationship of GO and Interpro terms. The KEGG annotation (Kanehisa and Goto, 2000) was done via the KAAS online server (Moriya et al., 2007) using the SBH method against the eukaryotic species set.

### Termite-specific genes

Some gene families were termite-specific and absent from the other investigated genomes. For these genes we performed functional enrichment analyses of GO and IPR (Interpro domain) annotation. *P*-values for significant difference were obtained by χ^2^-tests adjusted by FDR (false discovery rate). Similarly, we analyzed differences between the gene sets of *Z. nevadensis* und *M. natalensis* by comparing IPR annotation, KEGG pathways, and gene families. We constructed gene families for both genomes using Treefam (Li et al., 2006) and tested for differences in gene numbers using χ^2^-tests (or Fisher's exact test for small sample sizes). For gene families that were specific to *M. natalensis* and/or *Z. nevadensis*, we performed IPR enrichment analyses to obtain information on the putative functions of these genes.

### Repeat analyses

We used the *M. natalensis* and *Z. nevadensis* genome assemblies to perform repetitive sequence annotation. First, we did homologous repeat family annotation to identify transposable elements (TEs) using the TE database Repbase v17.06 (Jurka and Kapitonov, 2005) and the programs RepeatMasker (parameter –norna) and RepeatProteinMask v4.0.1 (http://www.RepeatMasker.org) (parameter –p 0.0001) (Smit et al., 1996-2010). *De-novo* repeat family annotation was done with PILER v1.0 (Edgar and Myers, 2005), LTRfinder v1.05 (Zhao and Wang, 2007) and RepeatModeler v1.05, (http://www.RepeatMasker.org) (Smit et al., 1996-2010) using default parameters. TEs identified by PILER were converted into TE families and aligned with Muscle v3.28 (Edgar, 2004) to obtain consensus sequences from the alignments. In order to reduce redundancy in the results of LTRfinder and PILER, an “all against all” BLASTn (*e*-value 1e-5) was performed. If sequences overlapped for more than 80% we kept the longer TE.

We combined the TE families with the consensus sequences of LTRfinder and PILER together with those identified using RepeatModeler to obtain the final TE sequence library for the two termites. All TE sequences were classified with RepeatClassifier in the RepeatModeler package against Repbase v17.06 (Jurka and Kapitonov, 2005) (Dataset S1). Finally, we used the *de novo* TE library to annotate all TEs in the two genomes and combined the results of homologous TE annotation and the *de novo* annotation. If there were overlapping annotations we kept the longer TE. In addition, we predicted tandem repeats using TRF finder (parameters settings: match = 2, mismatch = 7, delta = 7, PM = 80, PI = 10, Minscore = 50, and MaxPeriod = 12) (Benson, 1999). In total, the non-redundant repetitive sequences accounted for 27.8 and 45.9% of the *Z. nevadensis* and *M. natalensis* genome, respectively (Table 2, Dataset S1).

**Table 2:** **The number and length of each type of repetitive sequence**.

**Type**	***Macrotermes natalensis***			***Zootermopsis nevadensis***		
	**Number of repeats**	**Repeat length (bp)**	**Percentage of Genome (%)**	**Number of repeats**	**Repeat length (bp)**	**Percentage of Genome (%)**
TEs	525,847	118,593,042	10.12	307,278	53,444,656	10.83
LINE	1,027,017	237,020,224	20.22	171,545	32,495,416	6.59
LTR	33,435	6,864,870	0.59	10,625	1,980,023	0.40
Rolling Circle	12,725	3,630,172	0.31	2427	384,875	0.08
SINE	13,624	2,671,925	0.23	109,498	17,763,792	3.60
Unknown	535,062	121,413,841	10.36	115,074	22,629,266	4.59
Other	64	10,006	<0.001	3	185	<0.001
Simple repeat	390,741	40,059,393	3.42	88,333	9,086,992	1.84
Simple repeats	164,090	6504,930	0.55	113,670	4,338,842	0.88
Satellite and tandem repeats	221,634	74677,411	6.37	34,394	11,591,981	2.35
Non-redundant total	2,924,239	537,702,043	45.87	952,847	137,154,152	27.79

*Simple repeats are 2–5 bp repetitive units while longer satellite and tandem repeats have 6–40 bp. “Other” includes repeats that do not belong to any of the listed types, such as DNA-viruses or centromeric regions (listed in Table S1)*.

We also checked for Talua elements in both termite species, SINE elements that were first identified in termites (Luchetti, 2005; Luchetti and Mantovani, 2009). Talua reference sequences (Dataset S1) were mapped to the TE annotations using BLASTn (*e*-value 1e-5). If the alignment contained more than 50% of the Talua domain, the TE was considered to be a Talua containing TE. In total, we found 1575 and 4385 Talua containing TEs in the *Z. nevadensis* and *M. natalensis* genome, respectively.

## Results and discussion

### Genome architecture and repetitive sequences

A striking difference between ants and termites is that termite genomes are about three times larger (Table S1), which appears to be an ancestral cockroach characteristic (always several Gbs; Koshikawa et al., 2008). Termites actually have smaller genomes than cockroaches and it has been hypothesized that sociality was in fact associated with a reduction in genome size (Koshikawa et al., 2008). Yet the socially more complex *M. natalensis* has a genome size that is more than twice the genome size of *Z. nevadensis* (1.31 Gb vs. 562 Mb), which has the smallest genome known for any termite so far (Koshikawa et al., 2008). On the other hand, ant genome size appears to vary relatively little around an average of 300 Mb, with the largest ant genome published so far being 352 Mb (the red fire ant *Solenopsis invicta*) and smallest genome being 219 Mb (the Argentine ant *Linepithema humile*) (Table S2).

The two termite assemblies covered over 85% of the genomes, so any differences observed are unlikely to be related to the slightly fewer protein coding genes in *Z. nevadensis* (15,876 vs. 16,310 in *M. natalensis*). However, the *M. natalensis* genome contained a much higher proportion of repeat sequences (67.1 vs. 26.0% in *Z. nevadensis*) (Table 2). Subtracting these repeat sequences leads to comparable respective genome sizes of 367 and 365 Mb. Further genomic data will be needed to find out whether these ca. 365 Mbs represent a kind of “core genome” for termites and whether additional variation in genome size would then only be due to variation in repeat sequences. It will also be interesting to evaluate the first cockroach genomes to see whether their huge genomes (multiple Gbs) are associated with a higher number of coding or repeat sequences. In ants, genome-wide repeat content so far varies between 11.5 and 28.0% (Gadau et al., 2012) and no overall correlation with genome size appears to exist.

The *M. natalensis* genome had almost twice as many TEs (transposable elements) than the *Z. nevadensis* genome (45.9 vs. 27.8%; Table 2) and most of these were LINEs (long interspersed nuclear elements), which accounted for 20% of the *M. natalensis* genome (Table 2). According to the Rebase classification, most LINEs in *M. natalensis* resemble BovB retrotransposons, accounting for 16% of the genome, while LINEs contribute only ca. 3% in *Z. nevadensis* (Table 2). BovBs are relatively well known from vertebrates where they have a patchy distribution in squamates, monotremes, marsupials, ruminants, and several African mammals (Afrotheria), possibly as a consequence of horizontal gene transfer via reptile ticks (Walsh et al., 2013). In ruminants, part of one BovB LINE seems to have been recruited into a functional gene after duplication (Iwashita et al., 2006), but whether similar cooption processes may have occurred in termites remains to be explored.

The *M. natalensis* genome appears to have fewer SINEs (short interspersed nuclear elements) than the *Z. nevadensis* genome (3.6 vs. 0.2%). A new SINE retrotransposon, *Talua*, has recently been described for termites (Luchetti, 2005; Luchetti and Mantovani, 2009). It belongs to a new family of tRNA-derived elements that are very G+C-rich (55–60%) but makes up only a small proportion of the termite genomes (0.25 and 0.19% in *Z. nevadensis* and *M. natalensis*, respectively; Table S3). There are multi-copy TEs that are present in both termite genomes that do not resemble any known TEs. They may thus be novel termite-specific TEs, but additional termite and non-termite genomes will be needed to test this against a null hypothesis of being more general TEs that also occur in other hemimetabolous insects.

**Figure 3:**
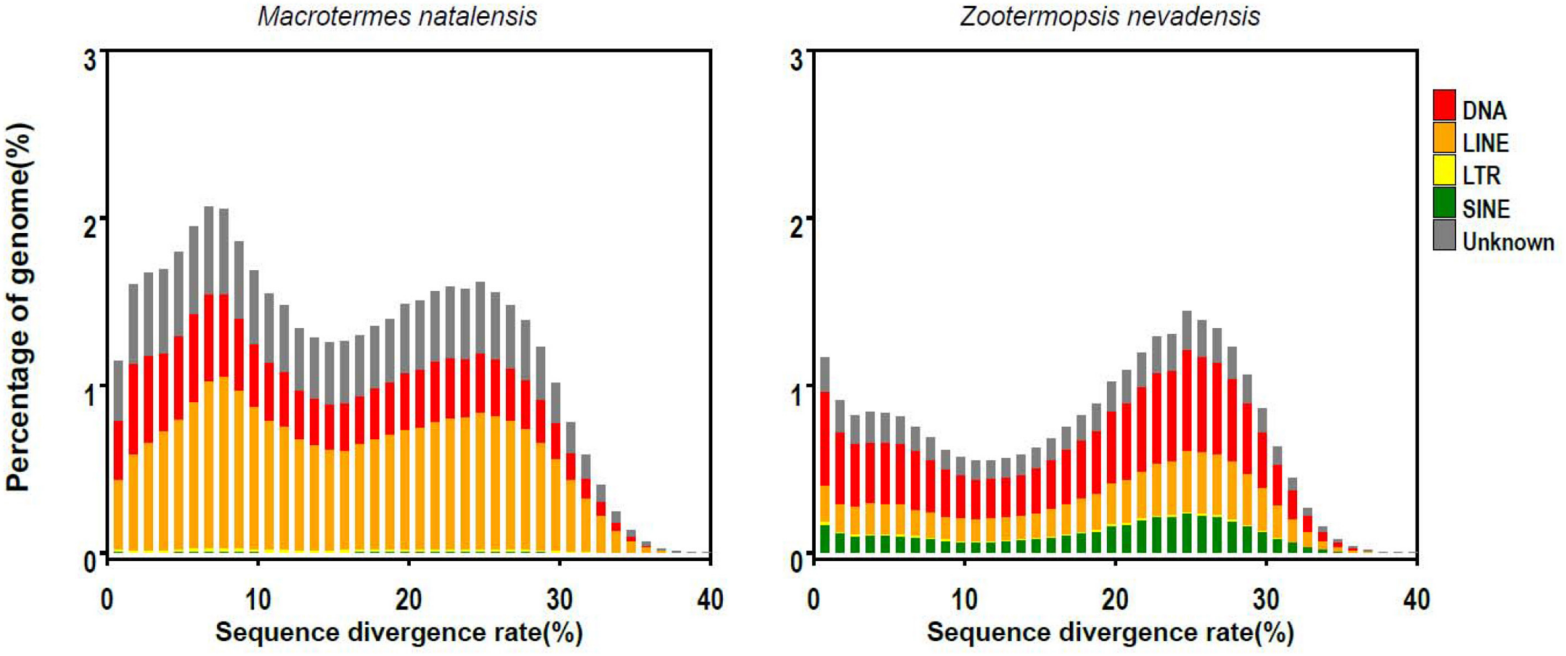
**The distribution of sequence divergence rates of transposable elements (TEs) as percentages of the genome size of *M. natalensis* (left) and *Z. nevadensis* (right)**.

TE sequence divergence (i.e., percentage of different base pairs) relative to TE consensus sequences showed a peak at about 25% for both *M. natalensis* and *Z. nevadensis* (Figure 3), but *M. natalensis* had an additional divergence rate peak at ca. 7~8% (Figure 3). This might indicate that the lineage leading to *M. natalensis* has undergone a genome expansion that multiplied TE copies and BovB retrotransposons, which could then explain why the *M. natalensis* genome is so much larger than the *Z. nevadensis* genome.

Consistent with the high prevalence of repeat sequences, IPR annotation results showed a functional enrichment of DNA/RNA cutting genes in termites (Ribonuclease H domain: 22 genes, Ribonuclease H-like domain: 26 genes, endonuclease/exonuclease/phosphatase: 26 genes) compared to other insects (Table S4). Strikingly, *M. natalensis* had at least twice as many of such transposon-related genes than *Z. nevadensis*, supporting the idea that selfish replicating elements played a major role in the evolution of termite genome architecture and size (Tables S5, S6).

Cluster analyses of caste-specific transcriptomes in *Z. nevadensis* revealed that several of these DNA/RNA-cutting genes are overexpressed in the nymphal stages (i.e., instars with wing buds) compared to all other stages and castes (Terrapon et al., 2014). Nymphs are individuals destined to develop into winged dispersing reproductives, suggesting that TE activity might be linked to maturation processes such as gonad development. Such functions remain speculative at this point, but would be consistent with TEs having been coopted to fulfill host functions and to play fundamental roles in epigenetic regulation in organisms as different as *Arabidopsis thaliana* plants, *Caenorhabditis elegans* worms, *Drosophila melanogaster* flies and *Mus musculus* house mice (e.g., Lippman et al., 2004; Slotkin and Martienssen, 2007; Fedoroff, 2012). Silenced TEs are often activated through stressful environmental conditions (Slotkin and Martienssen, 2007; Fedoroff, 2012). In wood-dwelling termites, such conditions may arise by reduced food availability or possibly parasite pressure inducing higher rates of nymphal (sexual dispersing) development (Lenz, 1994; Korb and Schmidinger, 2004; Korb and Fuchs, 2006). Hence, it may be interesting to test whether a similar link between TE activity and stressful conditions exists during nymphal development.

Whether TEs can also be linked with epigenetic regulation of gene expression through DNA methylation (Lippman et al., 2004; Slotkin and Martienssen, 2007; Fedoroff, 2012) remains to be seen. DNA methylation has been proposed to regulate caste differentiation (Kucharski et al., 2008; Elango et al., 2009; Gadau et al., 2012; Terrapon et al., 2014) and the complete epigenetic toolbox was indeed identified in *Z. nevadensis* with orthologs of *DNMT1* and *DNMT3* (Terrapon et al., 2014). However, in *M. natalensis* only *DNMT1* (and possibly *DNMT2*) could be confirmed, but not *DNMT3*.

### Communication

Termite-specific expansions for gene families were also found among chemoperception genes that are important for communication (Table S4). Given the disparate social systems of *Z. nevadensis* and *M. natalensis*, differences in expansions of such genes may be related to divergent communication systems. Chemoperception genes mainly comprise four families: Odorant receptors (ORs), gustatory receptors (GRs), ionotropic receptors (IRs), and odorant binding proteins. ORs mostly control for the specificity and sensitivity of insect olfaction. GRs are primarily involved in contact chemoperception and IRs belong to a recently discovered gene family for olfaction and gustation in *Drosophila* (Benton et al., 2009; Grosjean et al., 2011; Rytz et al., 2013). Odorant binding proteins primarily shuttle such compounds through the hydrophilic environment of the sensory lymph to the receptors.

The IR family is most consistently expanded in *Z. nevadensis*, representing the highest known value in insects (Terrapon et al., 2014). This IR number was between 4 and 10-fold higher in *Z. nevadensis* than in eusocial Hymenopterans, but the 80 intact GR genes remained within the overall range of 10–97 known from ants and honeybees (Zhou et al., 2012). The number of OR genes in *Z. nevadensis* was between one third and one half of the numbers normally found in the ants (Zhou et al., 2012), consistent with the lifestyle of wood-dwelling termites likely requiring lower levels of olfactory communication.

Overall, we found termite-specific enrichment in all four major gene families relating to olfaction (Table S4). Most IPR enrichment occurred in the ionotropic glutamate receptors that include IR genes (21). Significant enrichment was also found in ORs (7), GRs (7 TM chemoreceptor: 7), and various odorant-binding proteins (9, 7, 5). Direct comparison between *Z. nevadensis* and *M. natalensis* (Table S6) showed that *Z. nevadensis* had significantly more genes related to chemical communication than *M. natalensis* (Table S7). However, chemoperception genes are notoriously difficult to assemble and annotate (Terrapon et al., 2014), so this difference should be considered with caution, also because these genes were manually annotated in *Z. nevadensis* (with support from antennal RNAseq data), but automatically in *M. natalensis*. More work will therefore be needed before solid conclusions on the relative role of ORs in different termite species can be drawn.

### Immune defenses

Both termite species live in potentially pathogen-rich habitats. *Z. nevadensis* nests in decaying wood with abundant fungal growth that has probably selected for intensive allogrooming behaviors (Korb et al., 2012a). Also *M. natalensis* is potentially exposed to many pathogens both from its soil-nesting habitat and across its foraging range. *Macrotermes* species are known to protect their *Termitomyces* fungal symbiont from being overgrown by other fungi (Nobre et al., 2011) and termite-specific antimicrobial peptides (AMPs) have been described in another genus of fungus-growing termites (Lamberty et al., 2001).

**Figure 4:**
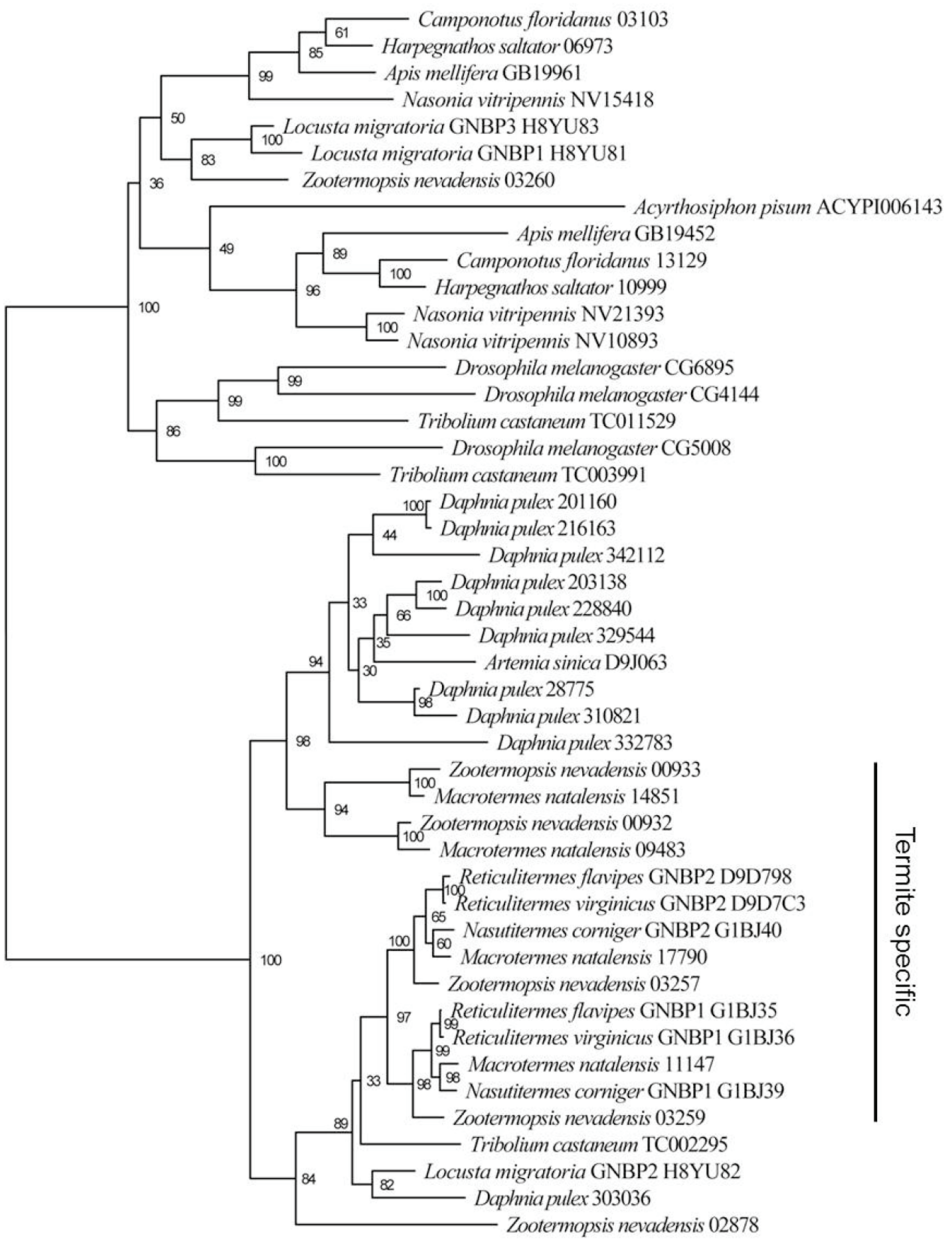
**Phylogeny of gram-negative binding proteins (GNBPs) constructed with PhylML v3.0 (LG substitution model with 100 bootstrap replicates) after alignment of the peptide sequences in ClustalW2**.

Relative to ants and other insects, we did not find enrichments for immune defense genes in the two termite genomes and neither were there substantial differences between the two termite genomes (Tables S4, S6). All of the immune-related pathways, including pattern recognition, signaling, and gene regulation (as described for *Drosophila melanogaster* and other insects; Hoffmann, 2003; Hultmark, 2003; Schmid-Hempel, 2005) are present in both termite genomes (Table S8). Only two differences are noteworthy (Table 3). First, *Z. nevadensis* has 6 gram-negative binding proteins (GNBPs), whereas only four of these were recovered in *M. natalensis*. These four GNBPs are all termite-specific (Figure 4) and some of them were previously shown to be under positive selection in several *Nasutitermes* species, especially in species with arboreal nests (Bulmer and Crozier, 2006). The *Macrotermes* genome seems to lack the insect-typical GNBP duplicate and one GNBP gene that has so far only been found in *Z. nevadensis* (Figure 4). Second, while AMPs were not enriched in either termite genome (Table S4), their identities were completely different with *Z. nevadensis* having 2 AMPs and *M. natalensis* having 3 other AMPs (Table 3, Table S8). *M. natalensis* has a termite-specific defensin-like gene termicin, a category of genes that seem to have duplicated repeatedly during the radiation of *Nasutitermes* termites (Bulmer and Crozier, 2004). After duplication, one copy seems to often be under strong selection, while the other evolves toward neutrality (Bulmer and Crozier, 2004; Bulmer et al., 2010). Also in the soil-foraging *Reticulitermes* species these genes seem to be under positive selection (Bulmer et al., 2010).

**Table 3:** **Gram-negative binding proteins (GNBPs) and anti-microbial-peptide (termicin) genes known from different termites**.

**Species**	**GNBP**	**Termicin**	**References**
*Z. nevadensis*	6 copies	0 copies	Terrapon et al., 2014
*Reticulitermes* sp.	Neutral	Positive selection	Bulmer et al., 2010
*Nasutitermes* spp. (Australia)	Positive selection in some species	Positive selection	Bulmer and Crozier, 2004, 2006
*Nasutitermes corniger*	Antifungal	?	Bulmer et al., 2009
*Pseudacanthotermes spiniger*	?	Yes	Lamberty et al., 2001
*M. natalensis*	4 copies	1 copy	Poulsen et al., 2014, this study

*GNBPs and termicins might serve complementary roles in fungal defense in termites. GNBPs might be more important in species with closed nests, whereas termicins seem to be under strong positive selection in foraging termites with subterranean nests. ?, unknown*.

In contrast to other insects where AMP production is normally induced, these genes seem to be constitutively expressed in fungus-growing termites, as has been shown for *Pseudacanthotermes spiniger* (Lamberty et al., 2001), which might be an adaptation to protect the symbiont against competing fungi. Termicin and other defensins (Table S8) were absent in *Z. nevadensis* but this species has GNBPs that are differentially expressed between castes (Terrapon et al., 2014) and may thus serve a similar function in protecting the nest from fungal infections. For the arboreal nesting termite *Nasutitermes corniger* it has been shown that GNBP2 has (1,3)-glucanase effector activity and functions as an antifungal agent (Bulmer et al., 2009). It is incorporated in the nest building material, where it cleaves and releases pathogenic components while priming termites for improved antimicrobial defense (Bulmer et al., 2009). Such a defensive strategy is likely to be most effective for termites with closed nests, consistent with positive selection on GNBP being most pronounced in *Nasutitermes* that live in arboreal nests (Bulmer and Crozier, 2006). Hence, antifungal stategies might differ in termites with different habitats; with GNBPs and termicin possibly playing complementary roles. This is supported by the fact that GNBPs in subterranean, foraging *Reticulitermes* species evolve neutrally while termicin was shown to have been under strong positive selection in these species (Table 3).

We can reject the possible alternative hypothesis that different defense strategies are linked to the gut symbionts that need different defense strategies to protect the symbiotic partner. As lower termites harbor protists as well as bacteria, while higher termites only have bacteria, we would then have expected higher termites having more AMPs and lower termites more GNBPs, but this is not the case because lower *Reticulitermes* termites have positively selected termicins. If there is an association between nesting habit and defense strategy, we expect that GNBPs are under positive selection in other wood-dwelling termites, and termicins are selected in soil-foraging termites. Additional genomic data, particularly for wood-dwelling termites, would be needed to validate this hypothesis.

Reduced numbers of immune defense genes were found in ants and the honeybee (Evans et al., 2006; Gadau et al., 2012) but also here there seems to be selection on some of the AMP genes. Similar to termicin, positive selection was detected on defensin in ants (Viljakainen and Pamilo, 2008), but this gene was not overexpressed after experimental fungal infections of leaf-cutting ant colonies, whereas two other AMPs were (Yek et al., 2013). This contrasts with dipterans (*Drosophila* and *Anopheles*) for which no evidence was found for positive selection on any AMPs (Sackton et al., 2007; Simard et al., 2007), but instead for immune recognition and signaling proteins (Schlenke and Begun, 2003; Jiggins and Kim, 2005; Sackton et al., 2007). This provides further support for the hypothesis that social insects have responded differently to selection pressure caused by microbial pathogens than solitary insects (Viljakainen and Pamilo, 2008).

### Mating biology

Compared to *M. natalensis*, the *Z. nevadensis* genome is enriched in genes that are related to male fertility/spermatogenesis (e.g., *KLHL10*) (Table 4, Table S7). This suggests that the co-expansion (and co-expression) of these genes in *Z. nevadensis* is not typical for termite sociality but rather taxon-specific. It might be linked to the seasonal reproduction of this temperate zone species where spermatogenesis is cyclically switched on and off, which contrasts with tropical *Macrotermes* males that produce offspring all year round. However, some members of two spermatogenesis-related gene families, seven-in-absentia (SINA) proteins and α-tubulins, do not show *Z. nevadensis*-specific expansions.

**Table 4:** **Number of genes related to spermatogenesis in *Z. nevadensis* and *M. natalensis* based on Pfam domains**.

**Protein families**	***Z. nevadensis***	***M. natalensis***
BTB-BACK-Kelch (KLHL10)	37	10
Kelch (KLHL1)	20	2
BTB+KELCH	6	1
BACK+KELCH	4	0
SINA (Seven-in-absentia)	33	17
Alpha tubulin	13	8
PKD (polycystin)	10	1

An alternative evolutionary explanation could be that males of wood-dwelling termites have low but consistent probabilities to face sperm competition when neighboring colonies merge after colony foundation. Such mergers are impossible in foraging termites where unrelated males never compete for inseminating the same queen (Boomsma, 2013). This hypothesis would predict no difference between temperate and tropical wood-dwelling termites, but a series of termite genomes will be needed to test these contentions.

### Symbiosis

The ancestral termite gut microbiota was derived from a cockroach ancestor, but major subsequent changes occurred, most notably when the higher termites evolved (Dietrich et al., 2014). The guts of the wood-dwelling termites are dominated by protists that appear to be primarily adapted to break down wood (Cleveland, 1923; Brugerolle and Radek, 2006), with complementary roles of bacteria that are often symbiotic with the gut-flagellates (Dietrich et al., 2014). The common ancestor of the evolutionarily derived Termitidae lost these flagellate symbionts so their gut microbiotas became dominated by bacteria, which may have facilitated their dietary diversification (Brune and Ohkuma, 2011; Dietrich et al., 2014). The single origin of fungiculture by the Macrotermitinae led to *Termitomyces* taking over primary plant decomposition and the gut microbiota shifting phylogenetically and functionally to perform complementary roles (Liu et al., 2013; Dietrich et al., 2014; Otani et al., 2014; Poulsen et al., 2014).

Changes in symbiont associations are tightly associated with termite life styles (for a recent review on termite gut symbionts, see Brune, 2014), but this may hardly induce structural genomic changes in the termite hosts, consistent with the similar gene repertoires for plant biomass decomposition found in the two termite genomes (Poulsen et al., 2014). A comparison of carbohydrate-active enzyme (CAZy) profiles of the two termite species showed a reduction in the absolute number of glycoside hydrolase enzymes (85) in *M. natalensis* compared to *Z. nevadensis* (97) (Table 5), but very similar relative abundances of specific enzyme families (Poulsen et al., 2014). Profile similarities suggest that plant-biomass decomposition genes may be ancestrally conserved across the termites, but additional termite genomes are needed to shed light on this. Such additional genomic work will need to be accompanied by enzyme function validations to test whether differences in absolute numbers reflect changes in the relative importance of termite-derived enzymes.

**Table 5:** **Number of glycoside hydrolases of different GH families identified in *Z. nevadensis* and *M. natalensis* (from Table S28; Poulsen et al., 2014)**.

**CAZy family**	***M. natalensis***	***Z. nevadensis***
GH1	11	7
GH2	5	4
GH9	4	6
GH13	8	9
GH15	1	1
GH16	4	5
GH18	12	14
GH20	6	8
GH22	3	3
GH27	1	2
GH29	1	2
GH30	2	2
GH31	4	6
GH35	2	1
GH37	3	3
GH38	3	3
GH39	1	1
GH47	4	5
GH56	1	1
GH63	1	1
GH74	1	1
GH79	1	2
GH84		1
GH85	1	1
GH89	1	1
GH99	1	1
GH109	2	5
GH119	1	1
Total	85	97

## Conclusion

Despite the striking differences in social complexity between *Z. nevadensis* and *M. natalensis* we did not find major differences in gene composition. The gene families underlying chemical communication seem not to be expanded in the more complex fungus-growing termite compared to *Z. nevadensis*. The major differences between the two termite genomes are related to genome architecture and the presence of transposons that can explain the much larger genome size of *M. natalensis*. Whether these ancestrally selfish elements have been domesticated for functions related to the increased social complexity of *M. natalensis* needs further work. Our comparison allowed us to generate hypotheses that can be tested with functional genomic studies and with more advanced comparative analyses as more termite genomes become available.

We have highlighted the contours of further testable predictions concerning TE number and genome size, male fertility, and habitat-specific disease pressure. For any next termite genome to be sequenced (Figure 1), authors should ask questions like: (1) Is the habitat of this (e.g., drywood) termite more disease-ridden than the habitat of a comparable dampwood termite such as *Z. nevadensis*? (2) Would this tropical new wood-dwelling termite have similar gene family expansions for male fertility as *Z. nevadensis*? (3) Has this arboreal higher (e.g., *Nasutitermes*) termite lost specific immune defenses that match the disease pressure of its habitat and is it equally burdened by TEs as *Macrotermes natalensis*?

While two genomes are a major achievement in some sense, these genomes also leave us with insufficient resolution to move much beyond the crude comparisons that we offer in this paper, because *Z. nevadensis* and *M. natalensis* differ in too many evolutionary and ecological factors (Table 1). It has also become clear from comparative ant genomics that gene expression mechanisms may be more informative than structural gene differences (Simola et al., 2013). Finally, apart from obtaining more termite genomes and population genomic studies on gene expression and signatures of selection, it will also be crucially important to obtain a *Cryptocercus* cockroach sister lineage genome and more distant outgroup genomes for non-social hemimetabolous insects. Many surprises will likely be waiting in the wings, as both the pea aphid and the body louse genomes turned out to be unusual because of the specialized feeding habits of these insects with or without symbionts.

### Conflict of interest statement

The Associate Editor Júrgen Rudolf Gadau declares that, despite being affiliated with the same institute and having collaborated with the author Jürgen Liebig, the review process was handled objectively. The authors declare that the research was conducted in the absence of any commercial or financial relationships that could be construed as a potential conflict of interest.
