# Supplementary material for: A genomic comparison of two termites with different social complexity
Source: Front Genet. 2015 Mar 4;6:9. doi: 10.3389/fgene.2015.00009 (PMC4348803; doi:10.3389/fgene.2015.00009)
Supplement: Supplementary file 1 [file Table1.DOCX]

**Table S1.** Comparison of the two termite genomes.

|  | ***Z. nevadensis*** | ***M. natalensis*** |
| --- | --- | --- |
| **Assembly accession number** | The whole genome shotgun project has been deposited in the Genbank nucleotide core database under the accession code AUST00000000. Genome sequence and annotation data are available at <http://www.termitegenome.org/?q=consortium_datasets>. A genome browser is available at <http://www.termitegenome.org/?q=browser>. | Raw reads have been deposited in the NCBI SRA database with accession nos. SRA069856 (*Macrotermes natalensis* genome), SRA071609 (*Termitomyces* genome), and SRA071613 (gut metagenomes). Assemblies are available from GigaScience: <http://dx.doi.org/10.5524/100055>. |
| **Average depth of coverage** | 98% | 63% |
| **Total genome size** | 562 MB | 1.309 GB |
| **Contig N_50_** | 20,030 bp | 15,640 bp |
| **Scaffold N_50_** | 740,215 bp | 1,997,143 bp |
| **% genomic G+C base composition** | 38% | 40% |
| **Protein-coding genes** | 15,876 (OGSv2.2) | 16,310 |
| **Total repeat content** | 26% | 67% |
