# Supplementary material for: A genomic comparison of two termites with different social complexity
Source: Front Genet. 2015 Mar 4;6:9. doi: 10.3389/fgene.2015.00009 (PMC4348803; doi:10.3389/fgene.2015.00009)
Supplement: Supplementary file 2 [file Table2.DOCX]

**Table S2.** Comparison of the ant genomes.

| **Species** | **Assembly size (bp)** | **contig N50 (bp)** | **scaffold N50 (bp)** |
| --- | --- | --- | --- |
| *Atta cephalotes* | 317,672,992 | 14,759 | 5,370,899 |
| *Acromyrmex echinatior* | 297,521,007 | 63,097 | 1,110,580 |
| *Camponotus floridanus* | 235,584,879 | 19,315 | 442,604 |
| *Harpegnathos saltator* | 298,683,418 | 37,977 | 601,965 |
| *Linepithema humile* | 219,806,118 | 34,298 | 1,451,070 |
| *Pogonomyrmex barbatus* | 235,355,905 | 10,707 | 816,513 |
| *Solenopsis incivta* | 352,687,102 | 18,125 | 720,578 |
