# Supplementary material for: A genomic comparison of two termites with different social complexity
Source: Front Genet. 2015 Mar 4;6:9. doi: 10.3389/fgene.2015.00009 (PMC4348803; doi:10.3389/fgene.2015.00009)
Supplement: Supplementary file 3 [file Table3.DOCX]

**Table S3.** Number of transposable elements that contain a Talua domain.

| **Species** | **Talua contain TE** | **Total length** | **Percentage of genome** |
| --- | --- | --- | --- |
| *Z. nevadensis* | 1575 | 1,245,818 bp | 0.25% |
| *M. natalensis* | 4385 | 2,205,498 bp | 0.19% |
