# Supplementary material for: A genomic comparison of two termites with different social complexity
Source: Front Genet. 2015 Mar 4;6:9. doi: 10.3389/fgene.2015.00009 (PMC4348803; doi:10.3389/fgene.2015.00009)
Supplement: Supplementary file 4 [file Table4.DOCX]

**Table S4.** IPR enrichment results of termite-specific genes

| **IPR ID** | **IPRTitle** | **P-value** | **Gene Number** |
| --- | --- | --- | --- |
| **IPR002156** | **Ribonuclease H domain** | 1.04E-24 | 22 |
| **IPR012337** | **Ribonuclease H-like domain** | 2.98E-18 | 26 |
| *IPR001320* | *Ionotropic glutamate receptor* | 2.82E-14 | 21 |
| *IPR023316* | *Pheromone/general odorant binding protein, PBP/GOBP, domain* | 9.17E-09 | 9 |
| *IPR006170* | *Pheromone/general odorant binding protein, PBP/GOBP* | 2.49E-06 | 7 |
| *IPR004117* | *Olfactory receptor, Drosophila* | 4.03E-06 | 7 |
| **IPR005135** | **Endonuclease/exonuclease/phosphatase** | 1.55E-05 | 26 |
| IPR010512 | Protein of unknown function DUF1091 | 3.25E-05 | 4 |
| *IPR006625* | *Insect pheromone/odorant binding protein PhBP* | 0.000298792 | 5 |
| *IPR012674* | *Calycin* | 0.000298792 | 5 |
| IPR008672 | Mitotic checkpoint | 0.000670959 | 3 |
| **IPR007087** | **Zinc finger, C2H2** | 0.001006972 | 23 |
| *IPR013604* | *7TM chemoreceptor* | 0.001021465 | 7 |
| IPR003591 | Leucine-rich repeat, typical subtype | 0.01194645 | 11 |
| **IPR015880** | **Zinc finger, C2H2-like** | 0.012243721 | 20 |
| **IPR013087** | **Zinc finger, C2H2-type/integrase, DNA-binding** | 0.040503149 | 18 |

IPRs in **bold** indicate **RNA/DNA binding & cutting** genes, IPRs in *italics* reflect genes associated with *chemical communication*
