# Supplementary material for: A genomic comparison of two termites with different social complexity
Source: Front Genet. 2015 Mar 4;6:9. doi: 10.3389/fgene.2015.00009 (PMC4348803; doi:10.3389/fgene.2015.00009)
Supplement: Supplementary file 5 [file Table5.DOCX]

**Table S5.** IPR enrichment results of genes specific in *Macrotermes natalensis*

| **IPR ID** | **IPRTitle** | **P-value** | **Gene Number** |
| --- | --- | --- | --- |
| **IPR000477** | **Reverse transcriptase** | 4.560E-144 | 221 |
| **IPR001888** | **Transposase, type 1** | 1.349E-99 | 198 |
| **IPR004875** | **DDE superfamily endonuclease, CENP-B-like**  **(probably also transposases)** | 2.222E-88 | 65 |
| **IPR012337** | **Ribonuclease H-like domain** | 5.517E-42 | 68 |
| **IPR004868** | **DNA-directed DNA polymerase, family B, mitochondria/virus** | 1.699E-34 | 39 |
| **IPR001584** | **Integrase, catalytic core** | 2.150E-32 | 35 |
| **IPR000358** | **Ribonucleotide reductase** | 5.084E-28 | 30 |
| **IPR023211** | **DNA polymerase, palm domain** | 1.984E-24 | 31 |
| **IPR001750** | **NADH:ubiquinone/plastoquinone oxidoreductase** | 2.623E-18 | 25 |
| **IPR006600** | **Pogo transposase / Cenp-B / PDC2, DNA-binding HTH domain** | 3.072E-16 | 22 |
| IPR013604 | 7TM chemoreceptor | 1.520E-15 | 23 |
| *IPR023780* | *Chromo domain* | 2.043E-15 | 21 |
| *IPR000953* | *Chromo domain/shadow* | 2.844E-14 | 23 |
| *IPR017452* | *GPCR, rhodopsin-like superfamily* | 1.438E-13 | 38 |
| *IPR016197* | *Chromo domain-like* | 9.363E-13 | 22 |
| **IPR006626** | **Parallel beta-helix repeat** | 1.378E-12 | 14 |
| *IPR000276* | *GPCR, rhodopsin-like, 7TM* | 7.545E-12 | 36 |
| IPR001694 | NADH:ubiquinone oxidoreductase, subunit 1/F420H2 oxidoreductase subunit H | 9.226E-10 | 10 |
| **IPR003591** | **Leucine-rich repeat, typical subtype** | 1.667E-08 | 30 |
| IPR000794 | Beta-ketoacyl synthase | 8.373E-07 | 14 |
| IPR014030 | Beta-ketoacyl synthase, N-terminal | 1.624E-05 | 10 |
| IPR010982 | Lambda repressor-like, DNA-binding | 2.162E-05 | 9 |
| IPR005312 | Protein of unknown function DUF1759 | 2.485E-05 | 8 |
| IPR016038 | Thiolase-like, subgroup | 4.500E-05 | 12 |
| *IPR005797* | *Cytochrome b/b6, N-terminal* | 1.572E-04 | 6 |
| **IPR006695** | **Centromere protein Cenp-B, DNA-binding domain 1** | 3.994E-04 | 6 |
| **IPR001611** | **Leucine-rich repeat** | 4.205E-04 | 24 |
| IPR009057 | Homeodomain-like | 4.373E-04 | 41 |
| *IPR005798* | *Cytochrome b/b6, C-terminal* | 9.262E-04 | 5 |
| **IPR006970** | **PT repeat** | 9.262E-04 | 5 |
| *IPR016175* | *Cytochrome b/b6* | 9.262E-04 | 5 |
| IPR003918 | NADH:ubiquinone oxidoreductase | 2.206E-03 | 4 |
| *IPR016174* | *Di-haem cytochrome, transmembrane* | 2.206E-03 | 4 |
| IPR001227 | Acyl transferase domain | 3.655E-03 | 7 |
| *IPR000298* | *Cytochrome c oxidase, subunit III* | 4.190E-03 | 3 |
| **IPR004603** | **DNA mismatch endonuclease vsr** | 4.190E-03 | 3 |
| IPR011677 | Domain of unknown function DUF1619 | 4.190E-03 | 3 |
| **IPR002492** | **Transposase, Tc1-like** | 5.053E-03 | 14 |
| IPR013831 | Esterase, SGNH hydrolase-type, subgroup | 8.588E-03 | 6 |
| **IPR000305** | **Excinuclease ABC, C subunit, N-terminal** | 1.319E-02 | 3 |
| IPR016039 | Thiolase-like | 1.979E-02 | 7 |
| **IPR001177** | **DNA helicase E1 protein, C-terminal, Papillomavirus** | 2.613E-02 | 2 |
| IPR001542 | Defensin, invertebrate/fungal | 2.613E-02 | 2 |
| **IPR003286** | **RNA-directed DNA polymerase, eukaryota** | 2.613E-02 | 2 |
| IPR004145 | Domain of unknown function DUF243 | 2.613E-02 | 2 |
| **IPR006579** | **Pre-C2HC** | 2.613E-02 | 2 |
| IPR006758 | Uncharacterised protein family B354L | 2.613E-02 | 2 |
| *IPR013833* | *Cytochrome c oxidase, subunit III, 4-helical bundle* | 2.613E-02 | 2 |
| IPR018416 | Na+/H+ exchanger, isoforms 3/9 | 2.613E-02 | 2 |
| IPR022048 | Protein of unknown function DUF3609 | 2.613E-02 | 2 |
| **IPR024445** | **Transposase, ISXO2-like** | 2.613E-02 | 2 |
| *IPR024791* | *Cytochrome c/ubiquinol oxidase subunit III* | 2.613E-02 | 2 |
| IPR001930 | Peptidase M1, alanine aminopeptidase/leukotriene A4 hydrolase | 3.286E-02 | 5 |
| IPR007652 | Alpha 1,4-glycosyltransferase domain | 3.905E-02 | 3 |
| IPR014782 | Peptidase M1, membrane alanine aminopeptidase, N-terminal | 4.138E-02 | 5 |

IPRs in **bold** indicate **Transposon/repeat** related genes, IPRs in *italics* reflect *cytochrome* related genes.
