# Supplementary material for: A genomic comparison of two termites with different social complexity
Source: Front Genet. 2015 Mar 4;6:9. doi: 10.3389/fgene.2015.00009 (PMC4348803; doi:10.3389/fgene.2015.00009)
Supplement: Supplementary file 6 [file Table6.DOCX]

**Table S6.** Top 20 significant IPR differences between *M. natalensis* and *Z. nevadensis*.

| **IPR** | **Function** | ***M. nat.*** | ***Z. nev.*** | **P-value** |
| --- | --- | --- | --- | --- |
| **IPR004875** | **Endonuclease/exonuclease/phosphatase** | **308** | 91 | 4.03E-28 |
| **IPR005135** | **Ribonuclease H-like domain** | **157** | 65 | 1.16E-09 |
| IPR023211 | Homeodomain-like | **227** | 115 | 3.00E-09 |
| **IPR001750** | **DDE superfamily endonuclease, CENP-B-like** | **86** | 3 | 4.87E-22 |
| **IPR000568** | **Integrase, catalytic core** | **61** | 14 | 4.17E-08 |
| IPR001584 | NADH:ubiquinone/plastoquinone oxidoreductase | **32** | 1 | 8.25E-09 |
| IPR012337 | Ribonucleotide reductase | **31** | 1 | 1.60E-08 |
| **IPR009057** | **DNA polymerase, palm domain** | **37** | 3 | 2.04E-08 |
| **IPR006626** | **Ribonuclease H domain** | **30** | 1 | 3.09E-08 |
| IPR000210 | Small GTPase superfamily, Ras type | **62** | 24 | 5.41E-05 |
| *IPR000358* | *Kelch repeat type 1* | 14 | **75** | 1.18E-11 |
| IPR002156 | Zinc finger, C3HC4 RING-type | 4 | **48** | 4.52E-11 |
| *IPR001370* | *BTB/POZ* | 50 | **114** | 2.41E-07 |
| IPR020849 | Galactose oxidase, beta-propeller | 6 | **38** | 4.14E-07 |
| *IPR006652* | *BTB/POZ-like* | 58 | **121** | 8.62E-07 |
| *IPR005312* | *BTB/Kelch-associated* | 19 | **59** | 3.45E-06 |
| *IPR005797* | *BTB/POZ fold* | 67 | **126** | 8.95E-06 |
| IPR018957 | EF-hand | 5 | **29** | 1.83E-05 |
| *IPR013069* | *Kelch-type beta propeller* | 15 | **48** | 2.04E-05 |

Most genes enriched in *M. natalensis* have IRP related to **RNA/DNA-cutting/transposons**. Most genes enriched in *Z. nevadensis* are related to *spermatogenesis*.
