# Supplementary material for: A genomic comparison of two termites with different social complexity
Source: Front Genet. 2015 Mar 4;6:9. doi: 10.3389/fgene.2015.00009 (PMC4348803; doi:10.3389/fgene.2015.00009)
Supplement: Supplementary file 7 [file Table7.DOCX]

**Table S7.** IPR enrichment results of genes specific in *Zootermopsis nevadensis*

| **IPR ID** | **IPRTitle** | **P-value** | **Gene Number** |
| --- | --- | --- | --- |
| **IPR006652** | **Kelch repeat type 1** | 3.002E-10 | 18 |
| IPR015916 | Galactose oxidase, beta-propeller | 4.878E-08 | 12 |
| *IPR004117* | *Olfactory receptor, Drosophila* | 7.422E-08 | 10 |
| IPR006578 | MADF domain | 7.422E-08 | 10 |
| *IPR013604* | *7TM chemoreceptor* | 1.252E-07 | 9 |
| **IPR015915** | **Kelch-type beta propeller** | 2.826E-06 | 11 |
| IPR013122 | Polycystin cation channel, PKD1/PKD2 | 2.826E-06 | 6 |
| IPR003915 | Polycystic kidney disease type 2 protein | 2.826E-06 | 5 |
| IPR001873 | Na+ channel, amiloride-sensitive | 5.350E-06 | 8 |
| IPR004210 | BESS motif | 1.536E-04 | 5 |
| IPR001810 | F-box domain, cyclin-like | 3.622E-04 | 9 |
| IPR006629 | LPS-induced tumor necrosis factor alpha factor | 3.622E-04 | 3 |
| IPR015925 | Ryanodine receptor-related | 2.785E-03 | 4 |
| IPR013568 | SEFIR | 2.967E-03 | 3 |
| IPR000618 | Insect cuticle protein | 4.347E-03 | 8 |
| IPR024571 | Domain of unknown function DUF3358 | 4.673E-03 | 4 |
| IPR009071 | High mobility group, superfamily | 4.835E-03 | 6 |
| IPR001930 | Peptidase M1, alanine aminopeptidase/leukotriene A4 hydrolase | 4.835E-03 | 4 |
| IPR006149 | EB domain | 4.835E-03 | 3 |
| IPR000905 | Peptidase M22, glycoprotease | 4.835E-03 | 2 |
| IPR002336 | Erythrocruorin | 4.835E-03 | 2 |
| IPR002579 | Methionine sulphoxide reductase B | 4.835E-03 | 2 |
| IPR006818 | Histone chaperone, ASF1-like | 4.835E-03 | 2 |
| IPR008717 | Noggin | 4.835E-03 | 2 |
| IPR013957 | Domain of unknown function DUF1777 | 4.835E-03 | 2 |
| IPR014306 | Hydroxyisourate hydrolase | 4.835E-03 | 2 |
| IPR015089 | Ubiquinol-cytochrome C reductase complex, 6.4kDa | 4.835E-03 | 2 |
| IPR017861 | Peptidase M22, glycoprotease, subgroup | 4.835E-03 | 2 |
| IPR023416 | Transthyretin/hydroxyisourate hydrolase, superfamily | 4.835E-03 | 2 |
| IPR001878 | Zinc finger, CCHC-type | 5.823E-03 | 5 |
| IPR000361 | FeS cluster biogenesis | 1.122E-02 | 2 |
| IPR003032 | Ryanodine receptor Ryr | 1.122E-02 | 2 |
| IPR004104 | Oxidoreductase, C-terminal | 1.122E-02 | 2 |
| IPR012292 | Globin, structural domain | 1.122E-02 | 2 |
| IPR016092 | FeS cluster insertion | 1.122E-02 | 2 |
| IPR006631 | Protein of unknown function DM4/12 | 1.990E-02 | 3 |
| IPR008893 | WGR domain | 1.990E-02 | 2 |
| IPR012919 | Sad1/UNC-like, C-terminal | 1.990E-02 | 2 |
| IPR000772 | Ricin B lectin domain | 2.256E-02 | 3 |
| IPR001734 | Sodium/solute symporter | 2.256E-02 | 3 |
| **IPR013069** | **BTB/POZ** | 2.649E-02 | 9 |
| IPR001766 | Transcription factor, fork head | 2.686E-02 | 3 |
| IPR000413 | Integrin alpha chain | 2.731E-02 | 2 |
| IPR000683 | Oxidoreductase, N-terminal | 2.731E-02 | 2 |
| IPR003958 | Transcription factor CBF/NF-Y/archaeal histone | 2.731E-02 | 2 |
| IPR013649 | Integrin alpha-2 | 2.731E-02 | 2 |
| *IPR001320* | *Ionotropic glutamate receptor* | 3.349E-02 | 6 |
| IPR001701 | Glycoside hydrolase, family 9 | 3.788E-02 | 2 |
| IPR008906 | HAT dimerisation | 3.788E-02 | 2 |
| IPR002557 | Chitin binding domain | 4.525E-02 | 5 |
| *IPR019594* | *Glutamate receptor, L-glutamate/glycine-binding* | 4.525E-02 | 3 |
| IPR007632 | Anoctamin/TMEM 16 | 4.750E-02 | 2 |
| IPR013519 | Integrin alpha beta-propellor | 4.750E-02 | 2 |
| IPR001611 | Leucine-rich repeat | 4.869E-02 | 8 |

IPRs in **bold** indicate genes related to **spermatogenesis**, IPRs in *italics* reflect genes associated with *chemical communication*
