# Supplementary material for: A genomic comparison of two termites with different social complexity
Source: Front Genet. 2015 Mar 4;6:9. doi: 10.3389/fgene.2015.00009 (PMC4348803; doi:10.3389/fgene.2015.00009)
Supplement: Supplementary file 8 [file Table8.DOCX]

**Table S8.** Immune related genes.

| **Gene name** | **Gene function/pathway** | ***M. natalensis* Gene ID** | ***Z. nevadensis* Gene ID** |
| --- | --- | --- | --- |
| Defensin | Effector/AMP | Mnat_08699 | - |
| Defensin | Effector/AMP | Mnat_08700 | - |
| Attacin | Effector/AMP | - | Znev_02297 |
| Diptericin | Effector/AMP | - | Znev_09129 |
| Termicin | Effector/AMP | Mnat_18061 | - |
| Lysozyme C-type | Lysozyme | - | Znev_03738 |
|  | Lysozyme | Mnat_05526 | Znev_17477 |
|  | Lysozyme | - | Znev_11184 |
| Lysozyme I-type | Lysozyme | - | Znev_07418 |
|  | Lysozyme | Mnat_08863 | Znev_01125 |
|  | Lysozyme | - | Znev_11158 |
|  | Lysozyme | - | Znev_07419 |
| GNBP | Pattern Recognition | - | Znev_03260 |
|  | Pattern Recognition | - | Znev_02878 |
|  | Pattern Recognition | Mnat_17790 | Znev_03257 |
|  | Pattern Recognition | Mnat_11147 | Znev_03259 |
|  | Pattern Recognition | - | Znev_00932 |
|  | Pattern Recognition | Mnat_14851 | Znev_00933 |
| PGRP | Pattern Recognition | - | Znev_07518 |
|  | Pattern Recognition | Mnat_09373 | Znev_09910 |
|  | Pattern Recognition | Mnat_15826 | Znev_08618 |
|  | Pattern Recognition | Mnat_09372 | Znev_09909 |
|  | Pattern Recognition | - | Znev_07984 |
|  | Pattern Recognition | - | Znev_01249 |
| 18wheeler | Toll receptor | Mnat_08472 | Znev_10053 |
| Toll/Tollo | Toll receptor | Mnat_09387 | Znev_00888 |
|  | Toll receptor | Mnat_08454 | Znev_10041 |
|  | Toll receptor | Mnat_08462 | Znev_10044 |
| Toll | Toll receptor | Mnat_13328 | Znev_13969 |
| Toll or LRR | Toll receptor | Mnat_16873 | Znev_05966 |
| Toll-9 | Toll receptor | Mnat_08454 | Znev_10923 |
| Toll-like receptor | Toll receptor | Mnat_13268 | Znev_01370 |
| Tollip (Toll-interacting protein) | TOLL pathway | Mnat_05224 | Znev_07741 |
| ECSIT (signal intermediate in Toll pathway) | TOLL pathway | Mnat_03270 | Znev_07957 |
| Pelle | TOLL pathway | Mnat_10544 | Znev_09453 |
| Tube | TOLL pathway | Mnat_17672 | Znev_05846 |
| TRAF | TOLL pathway | Mnat_12133 | Znev_17913 |
|  | TOLL pathway | - | Znev_11985 |
|  | TOLL pathway | - | Znev_15253 |
| Spaetzle | TOLL pathway | - | Znev_00635 |
|  | TOLL pathway | Mnat_09529 | Znev_04528 |
| Spaetzle-like | TOLL pathway | - | Znev_11366 |
|  | TOLL pathway | Mnat_15218 | Znev_10323 |
|  | TOLL pathway | - | Znev_10324 |
| DIF/DORSAL | TOLL pathway | Mnat_11843 | Znev_07478 |
| Easter (Spaetzle-Processing enzyme) | TOLL pathway | Mnat_16923 | Znev_10162 |
| JNK-interacting SapK | TOLL pathway | Mnat_02656 | Znev_15407 |
| Relish (NF-Kappa-B) | NF-K-B-related | Mnat_03065 | Znev_11193 |
| C-Jun/JNK | NF-K-B-related | Mnat_12693 | Znev_00650 |
| Mpk2 | NF-K-B-related | Mnat_00458 | Znev_02213 |
| Cactus | NF-K-B-related | Mnat_16833 | Znev_07020 |
| NF-kappa-B inhibitor alpha | NF-K-B-related | Mnat_02159 | Znev_09660 |
| NF-kappa-B inhibitor-like | NF-K-B-related | - | Znev_03760 |
| Kappa-B-ras (NF-kappa-B inhibitor alpha-interacting) | NF-K-B-related | Mnat_08997 | Znev_06522 |
| NF-kappa-B-repressing factor | NF-K-B-related | Mnat_11635 | Znev_05897 |
| STAT | JAK-STAT pathway | Mnat_03453 | Znev_16675 |
| Cytokine receptor | JAK-STAT pathway | Mnat_02248 | Znev_09344 |
| JAK pathway STAM | JAK-STAT pathway | Mnat_12055 | Znev_00434 |
| JAK/hopscotch | JAK-STAT pathway | Mnat_00665 | Znev_10639 |
| IKB (I-Kappa-B) | IMD pathway | Mnat_12011 | Znev_09963 |
| IMD (immune deficiency) | IMD pathway | Mnat_01792 | Znev_02405 |
| FAS-assocaited factor (TNFRSF6) | IMD pathway | Mnat_03695 | Znev_08114 |
| Optineurin (NF-K-B modulator) | IMD pathway | Mnat_14701 | Znev_04648 |
| MAPKKK (TAK1) | IMD pathway | Mnat_00234 | Znev_09904 |
| MAPKKK | IMD pathway | Mnat_07077 | Znev_12168 |
| MYLIP (defense repressor) | IMD pathway | Mnat_09849 | Znev_02112 |
| NIK + IKBKB-BP (TRAF-like) | IMD pathway | Mnat_07994 | Znev_04156 |
| prophenoloxidase | PO-related | Mnat_06018 | Znev_05598 |
| Hemocyanin | PO-related | Mnat_10414 | Znev_04925 |
|  | PO-related | Mnat_10414 | Znev_04926 |
| Coagulation factor XI | PO-related | Mnat_01748 | Znev_16656 |
| Prophenoloxidase-activating enzyme 2 | PO-related | Mnat_16927 | Znev_18221 |
| TEP1 | Thioester-containing protein | Mnat_13155 | Znev_02879 |
| TEP2 | Thioester-containing protein | Mnat_00655 | Znev_18586 |
| TEP3 | Thioester-containing protein | Mnat_00634 | Znev_13964 |
| TEP4 | Thioester-containing protein | Mnat_00634? | Znev_05513 |
| Dual oxidase/heme peroxidase | Peroxidase | Mnat_05884 | Znev_17480 |
| Dual oxidase/heme peroxidase | Peroxidase | Mnat_05884? | Znev_00592 |
| Peroxidasin/Chorion peroxidase | Peroxidase | Mnat_07429 | Znev_16904 |
| Peroxidasin/Chorion peroxidase | Peroxidase | Mnat_09436 | Znev_01987 |
| Peroxidasin/Chorion peroxidase | Peroxidase | - | Znev_16752 |
| Peroxidasin/Chorion peroxidase | Peroxidase | Mnat_10052 | Znev_09888 |
| Peroxidasin/Chorion peroxidase | Peroxidase | Mnat_13664 | Znev_02993 |
| Peroxidasin/Chorion peroxidase | Peroxidase | - | Znev_16996 |
| Peroxidase | Peroxidase | - | Znev_03574 |
| Peroxidase | Peroxidase | Mnat_03969 | Znev_03575 |
| ATG2 (Autophagy-related protein 2) | Autophagy | Mnat_01012 | Znev_14004 |
| ATG2 | Autophagy | - | Znev_11462 |
| ATG3 | Autophagy | Mnat_10861 | Znev_06264 |
| ATG4B | Autophagy | Mnat_09510 | Znev_05555 |
| ATG4D | Autophagy | Mnat_02222 | Znev_10705 |
| ATG5 | Autophagy | Mnat_01056 | Znev_12390 |
| ATG6 (Beclin) | Autophagy | Mnat_05003 | Znev_07723 |
| ATG6 (Beclin) | Autophagy | - | Znev_17966 |
| ATG7 | Autophagy | Mnat_04612 | Znev_17566 |
| ATG7 | Autophagy | - | Znev_04460 |
| ATG8 (Gabarap) | Autophagy | Mnat_12800 | Znev_13859 |
| ATG9 | Autophagy | Mnat_08909 | Znev_01174 |
| ATG10 | Autophagy | Mnat_10685 | Znev_06925 |
| ATG12 | Autophagy | Mnat_05724 | Znev_07412 |
| ATG16L1 | Autophagy | Mnat_01768 | Znev_10009 |
| ATG16L1 | Autophagy | Mnat_01765 | Znev_10012 |
| RB1-inducible coiled-coil | Autophagy | Mnat_15881 | Znev_02531 |
| ULK2 (unc-51-like kinase 2) | Autophagy | Mnat_13218 | Znev_10659 |
| ULK3 | Autophagy | Mnat_12946 | Znev_04866 |
| Wipi1 (WD repeat domain phosphoinositide-interacting protein 1) | Autophagy | Mnat_02779 | Znev_09191 |
| Wipi3 | Autophagy | Mnat_08196 | Znev_13104 |
| Wipi4 | Autophagy | Mnat_17873 | Znev_10905 |
| WD repeat-containing protein 65 | Autophagy | Mnat_07247 | Znev_02990 |
| APAF-1 (apoptotic protease activating factor 1) | Apoptosis | Mnat_07027 | Znev_00248 |
| APAF-2 | Apoptosis | Mnat_17875 | Znev_00246 |
| BAX (Apoptosis inhibitor) | Apoptosis | Mnat_16520 | Znev_15412 |
| Ice (effector caspase-1) | Apoptosis | Mnat_04578 | Znev_13497 |
| Effector caspase | Apoptosis | - | Znev_14304 |
| Effector caspase | Apoptosis | Mnat_10647 | Znev_17676 |
| Effector caspase | Apoptosis | Mnat_05242 | Znev_18362 |
| Effector caspase | Apoptosis | Mnat_02324 | Znev_08436 |
| DREDD/Nedd2 | Apoptosis | Mnat_01071 | Znev_13406 |
| DCN1-like protein | Apoptosis | Mnat_15698 | Znev_00522 |
| Fas (TNFRSF6)-associated via death domain | Apoptosis | - | Znev_06474 |
| Ankyrin repeat and death domain-containing protein | Apoptosis | Mnat_09713 | Znev_09041 |
| Galectin | Lectin | - | Znev_07311 |
| Galectin | Lectin | Mnat_12476 | Znev_04392 |
| CTL | C-Lectin | - | Znev_00043 |
| CTL | C-Lectin | - | Znev_01827 |
| CTL | C-Lectin | Mnat_09515 | Znev_05559 |
| CTL | C-Lectin | - | Znev_16861 |
| CTL | C-Lectin | - | Znev_05556 |
| CTL | C-Lectin | - | Znev_00446 |
| CTL (Macrophage mannose receptor 1) | C-Lectin | Mnat_12207 | Znev_14909 |
| CTL (sushi, von Willebrand factor type A, EGF and pentraxin domain-containing protein) | C-Lectin | Mnat_08501 | Znev_08675 |
| CTL (sushi, von Willebrand factor type A, EGF and pentraxin domain-containing protein) | C-Lectin | Mnat_14624 | Znev_03178 |
| NPC2-like | ML superfamily (MD-2-related lipid-recognition) | Mnat_02054 | Znev_00122 |
| MPA2 allergen | ML superfamily | Mnat_07637 | Znev_11387 |
| MPA2 allergen | ML superfamily | - | Znev_11388 |
| MDL1 | ML superfamily | - | Znev_17997 |
| MDL2 | ML superfamily | Mnat_07636 | Znev_11389 |
| SCARA (Scavenger receptor class A-like) | Scavenger Receptor A | Mnat_05241 | Znev_09392 |
| SCARA | Scavenger Receptor A | - | Znev_09907 |
| SCARA | Scavenger Receptor A | - | Znev_03412 |
| SCARA | Scavenger Receptor A | Mnat_01920 | Znev_12289 |
| SRCR (scavenger receptor class A-like, cysteine-rich) | Scavenger Receptor A | Mnat_14222 | Znev_12950 |
| SRCR | Scavenger Receptor A | Mnat_00402 | Znev_12911 |
| SCARB (scavenger receptor class B), croquemort type | Scavenger Receptor B | Mnat_13543 | Znev_01894 |
| SCARB, croquemort type | Scavenger Receptor B | Mnat_04550 | Znev_01929 |
| SCARB, croquemort type | Scavenger Receptor B | - | Znev_17151 |
| SCARB | Scavenger Receptor B | - | Znev_09663 |
| SCARB | Scavenger Receptor B | Mnat_02161 | Znev_09665 |
| SCARB | Scavenger Receptor B | Mnat_02162 | Znev_09668 |
| SCARB | Scavenger Receptor B | - | Znev_17970 |
| SCARB | Scavenger Receptor B | - | Znev_05148 |
| SCARB | Scavenger Receptor B | - | Znev_14227 |
| SCARB | Scavenger Receptor B | Mnat_03839 | Znev_05147 |
| LDL receptor | LDL | Mnat_10290 | Znev_11465 |
| LDL receptor | LDL | Mnat_10291 | Znev_11466 |
| LDL receptor | LDL | - | Znev_11467 |
| T-cell immunomodulatory protein |  | Mnat_05563 | Znev_04939 |
| IG-binding protein | TOR pathway | Mnat_05936 | Znev_11186 |
| IG-domain containing | IG superfamily | Mnat_07315 | Znev_15173 |
| serine protease inhibitor | Serpin | - | Znev_11231 |
|  | Serpin | - | Znev_03219 |
|  | Serpin | - | Znev_11099 |
|  | Serpin | Mnat_09728 | Znev_01881 |
|  | Serpin | - | Znev_11098 |
|  | Serpin | - | Znev_11095 |
|  | Serpin | - | Znev_07264 |
|  | Serpin | - | Znev_02826 |
|  | Serpin | - | Znev_06875 |
|  | Serpin | - | Znev_11096 |
|  | Serpin | - | Znev_10043 |
|  | Serpin | - | Znev_02802 |
|  | Serpin | Mnat_08764 | Znev_05728 |
| Leukocyte elastase inhibitor | Serpin | Mnat_09858 | Znev_06230 |
| cSP (serine protease stubble) | cSP | Mnat_08814 | Znev_16873 |
|  | cSP | - | Znev_17166 |
|  | cSP | Mnat_00200 | Znev_12938 |
|  | cSP | - | Znev_07739 |
|  | cSP | Mnat_13391 | Znev_05138 |
|  | cSP | Mnat_08812 | Znev_03586 |
|  | cSP | Mnat_08813 | Znev_03587 |
|  | cSP | - | Znev_03593 |
|  | cSP | - | Znev_06101 |
|  | cSP | Mnat_11055 | Znev_09866 |
|  | cSP | Mnat_01747 | Znev_12298 |
|  | cSP | Mnat_05819 | Znev_05193 |
| Superoxide dismutase | SOD | - | Znev_15634 |
|  | SOD | Mnat_07013 | Znev_12756 |
|  | SOD | Mnat_06159 | Znev_11634 |
